# Dietary phytate primes epithelial antibacterial immunity in the intestine

**DOI:** 10.3389/fimmu.2022.952994

**Published:** 2022-10-19

**Authors:** Seika Hashimoto-Hill, Luisa Colapietro, Vivienne Woo, Simona Antonacci, Jordan Whitt, Laura Engleman, Theresa Alenghat

**Affiliations:** Division of Immunobiology, and Center for Inflammation and Tolerance, Cincinnati Children’s Hospital Medical Center and the University of Cincinnati College of Medicine, Cincinnati, OH, United States

**Keywords:** diet, microbiota, intestine, innate immunity, metabolite

## Abstract

Although diet has long been associated with susceptibility to infection, the dietary components that regulate host defense remain poorly understood. Here, we demonstrate that consuming rice bran decreases susceptibility to intestinal infection with *Citrobacter rodentium*, a murine pathogen that is similar to enteropathogenic *E. coli* infection in humans. Rice bran naturally contains high levels of the substance phytate. Interestingly, phytate supplementation also protected against intestinal infection, and enzymatic metabolism of phytate by commensal bacteria was necessary for phytate-induced host defense. Mechanistically, phytate consumption induced mammalian intestinal epithelial expression of STAT3-regulated antimicrobial pathways and increased phosphorylated STAT3, suggesting that dietary phytate promotes innate defense through epithelial STAT3 activation. Further, phytate regulation of epithelial STAT3 was mediated by the microbiota-sensitive enzyme histone deacetylase 3 (HDAC3). Collectively, these data demonstrate that metabolism of dietary phytate by microbiota decreases intestinal infection and suggests that consuming bran and other phytate-enriched foods may represent an effective dietary strategy for priming host immunity.

## Introduction

Intestinal infections pose a serious threat to public health worldwide, with reported cases exceeding two billion and over one million deaths each year (1). These infections are a leading cause of death among children, and surviving children can suffer from long-term health consequences such as delayed growth and vaccine failure (2, 3). Given that the intestinal mucosa is a primary site of exposure for multiple pathogens, deciphering the pathways that guide intestinal defense is critical for developing new approaches for treating and preventing infection.

Nutrition affects various aspects of physiology, including the immune system, as demonstrated by a strong correlation between malnutrition and infection morbidity (4, 5). Dietary components such as carbohydrates, lipids, amino acids, vitamins, and minerals have each been implicated in the regulation of host defense against pathogens (6, 7). For example, consumption of a high-fat diet increases disease in rodents following infection with *C. difficile, C. rodentium*, or *Listeria monocytogenes* (8–10). Trillions of commensal microbes reside in the mammalian intestine and are collectively referred to as the microbiota. Increasing evidence indicates that the metabolism of dietary nutrients by resident commensal microbes alters host physiology (11). Microbial metabolism results in the production of metabolites and small-molecule intermediates that regulate the symbiotic relationship between the microbiota and host (12). For example, microbiota-derived metabolites such as short-chain fatty acids (SCFAs) (13–15), indoles (16, 17), secondary bile acids (18, 19), and siderophores (20) have been described to protect during mouse models of infection. However, despite evidence linking nutrition and immunity, mechanistic insights needed to guide how diet can be modified to optimize host immunity are limited (11).

Intestinal epithelial cells (IECs) reside at the direct interface between the host and commensal microbes and, therefore, carry the potential to critically respond to signals from the diet, microbiota, and luminal metabolites (21–23). IECs provide the first line of defense against invading pathogens with constitutive expression of defense molecules, including antimicrobial peptides (AMPs), reactive oxygen species, and mucins (24, 25). Germ-free and microbiota depletion studies have shown that commensal microbial signals are required for basal expression of many AMPs (26, 27). IEC expression of the AMP regenerating islet-derived protein 3γ (Reg3γ) requires signaling through Toll-like receptors (TLRs) and other microbiota-sensitive pathways (28). In addition, nutritional regulation of AMP expression has also been suggested, and the timing of food intake can significantly alter IEC expression of AMPs (29, 30). However, the dietary factors that cooperate with the microbiota to prime IEC-mediated defense are not well known.

Phytate is in various foods, including bran, legumes, seeds, and nuts, and is enriched in diets such as vegetarian and Mediterranean (31–33). Mineral-chelating properties of phytate have been historically discussed in relation to rickets, which is caused by reduced calcium and phosphorus availability (34). Although phytate is no longer considered a primary pathogenic factor in rickets, absorption interference of iron, zinc, and other minerals with high doses of phytate could occur in the context of mineral deficiency, leading phytate to commonly be termed an anti-nutrient (35). However, diets containing phytate in combination with sufficient minerals do not present with disorders related to trace mineral absorption (33).

Rice bran is an abundant by-product generated during rice milling that has been gaining popularity as a food supplement over the past ten years (36, 37). Here, we discovered that ingestion of rice bran reduced infection burden and related pathology in a murine *Citrobacter rodentium* infection, the mouse model for enteropathogenic and enterohemorrhagic *E. coli* infection prevalent in the human population. Rice bran contains high amounts of phytate. Interestingly, phytate supplementation similarly protected against intestinal infection. This diet-induced protection was dependent on microbial digestion of phytate and the production of phytate metabolites. Mechanistically phytate induced STAT3 activation and the downstream defense pathway in the IECs. Phytate-induced STAT3 activation was mediated by the metabolite-sensitive enzyme HDAC3 in an IEC-intrinsic manner. Collectively, these findings reveal new diet-microbiota interactions that promote innate intestinal immunity and mammalian defense against infection.

## Results

### Consuming rice bran decreases susceptibility to *C. rodentium* infection

**Figure 1:**
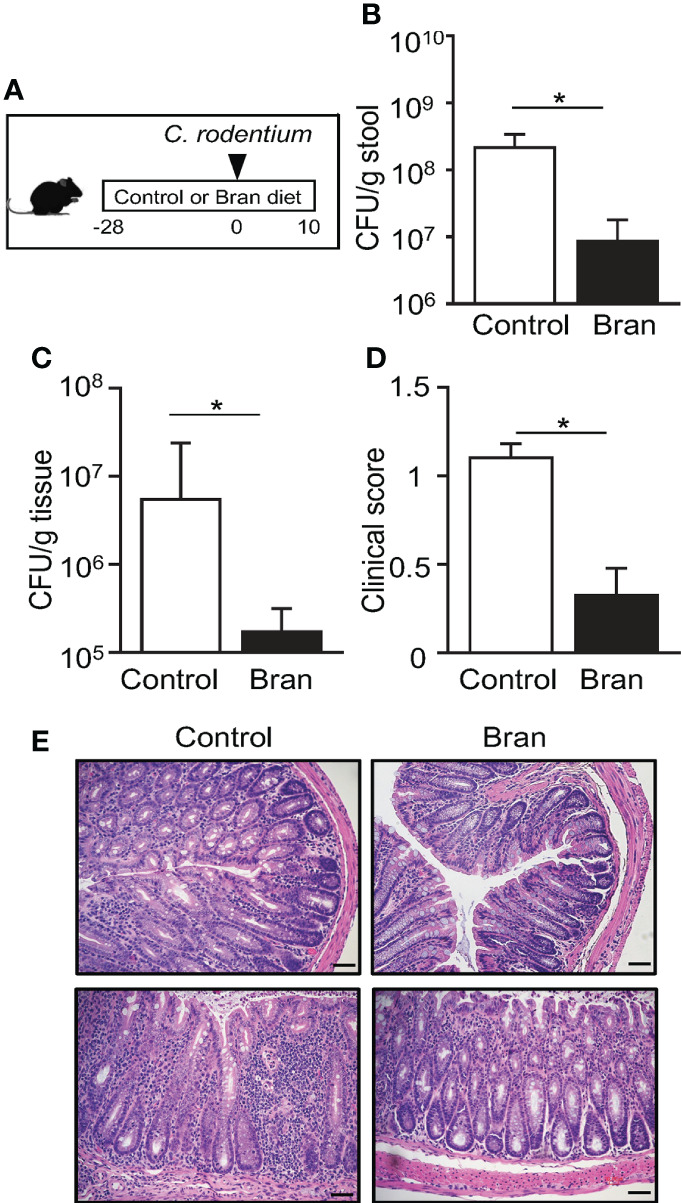
Consuming rice bran decreases susceptibility to *C. rodentium* infection. **(A)** Experimental approach. **(B)** Colony-forming units (CFUs) of *C. rodentium* in stool of infected control- or 20% rice bran diet-fed mice, normalized to sample weight, days 3 post-infection. **(C)** CFUs of *C. rodentium* in the colon tissues, day 10 post-infection. **(D)** Clinical scores representing severity of diarrhea, day 10 post-infection. **(E)** Histological staining of the colon tissues of infected mice, day 10 post-infection. Scale bars: 10 μm. Data are representative of 2-3 independent experiments. n = 4 per group. Results are mean ± SEM. ^*^ *p* < 0.05.

Rice bran has been proposed to provide broad health benefits ranging from weight loss, cancer prevention, and protection from infection (37–40), provoking the hypothesis that rice bran may alter susceptibility to pathogens like *E. coli*. To test this, littermate mice were exclusively fed a custom diet containing 20% rice bran or a matched control diet for four weeks, and then infected with *Citrobacter rodentium* (**Figure 1A**). *C. rodentium* is a murine intestinal pathogen with similar pathogenesis to enteropathogenic and enterohemorrhagic *E. coli*, two leading causes of food-borne illnesses in humans (41–44). Interestingly, mice ingesting rice bran exhibited significantly reduced *C. rodentium* in the intestinal lumen (**Figure 1B**) and colonic tissue (**Figure 1C**) post-infection compared to control diet-fed mice (**Figures 1B, C**). Differences in pathogen burden were observed by day 3 post-infection, suggesting that bran diet decreased initial *C. rodentium* colonization and replication in the large intestinal mucosa. Consistent with decreased infection, *C. rodentium*-induced changes in stool consistency were less severe in the rice bran-fed mice compared to control diet-fed mice (**Figure 1D**). Pathological features of *C. rodentium*, such as colonic epithelial hyperplasia and leukocyte infiltration, were observed in control diet-fed mice (**Figure 1E**). However, these histologic features of *C. rodentium* infection were diminished in bran-fed mice (**Figure 1E**). Taken together, these findings suggest that a component of rice bran may decrease susceptibility to bacterial infection.

### The natural component phytate in rice bran protects mice against *C. rodentium*

**Figure 2:**
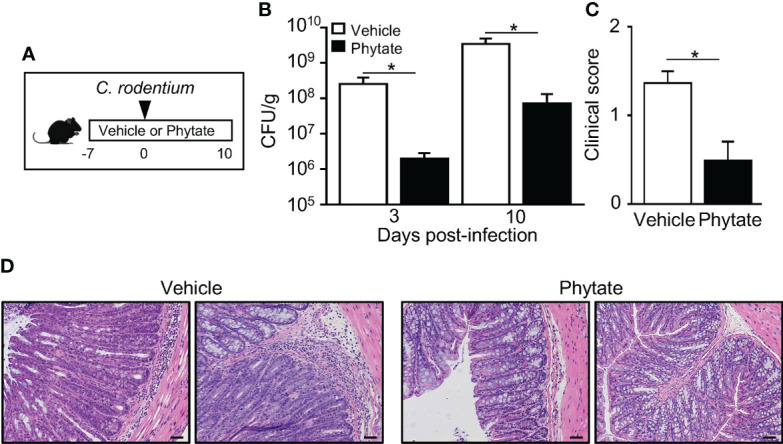
The natural component phytate in rice bran protects mice against *C. rodentium*. **(A)** Experimental approach. **(B)** CFUs of *C. rodentium* in stool of infected vehicle- or 2% phytate-treated mice, normalized to sample weight, days 3 and 10 post-infection. **(C)** Clinical scores representing severity of diarrhea, day 10 post-infection. **(D)** Histological staining of colon tissues of infected mice, day 10 post-infection. Scale bars: 10 μm. Data are representative of 2-3 independent experiments. n=4 per group. Results are mean ± SEM. ^*^ *p* < 0.05.

Rice bran is naturally enriched with phytate (31). Therefore, to test whether phytate itself alters *C. rodentium* infection, control mice and mice receiving 2% phytate were compared during infection (**Figure 2A**). Interestingly, similar to the outcome with rice bran ingestion, phytate consumption significantly decreased pathogen burden post-infection (**Figure 2B**). Consistent with decreased pathogen levels, clinical symptoms of infection were also less severe in phytate-treated mice relative to controls (**Figure 2C**). To examine pathology induced by *C. rodentium* infection, colonic tissue was examined following peak infection. As expected, *C. rodentium* induced colonic epithelial hyperplasia and leukocyte infiltration in control mice (**Figure 2D**). However, infection-associated pathology was reduced in mice fed phytate, recapitulating findings that occur with the rice bran diet (**Figure 1**). Collectively these data demonstrate that consuming phytate is sufficient to promote protection against intestinal bacterial infection.

### Metabolism of phytate by commensal bacteria mediates protection against infection

**Figure 3:**
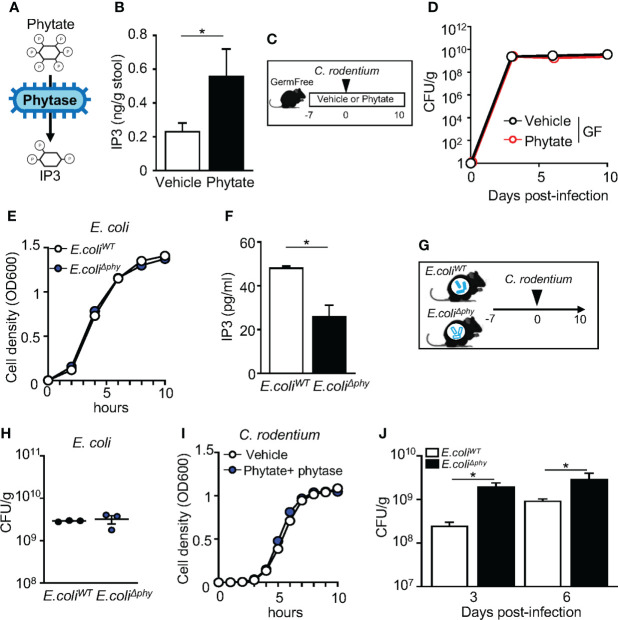
Metabolism of phytate by commensal bacteria mediates protection against infection. **(A)** Schematic of phytate metabolism by microbial phytase. **(B)** Inositol trisphosphate (IP3) concentration in fecal samples collected from vehicle- or 2% phytate-treated mice, normalized to sample weight. **(C)** Experimental approach. **(D)** CFUs of *C. rodentium* in stool of infected vehicle- or 2% phytate-treated GF mice, normalized to sample weight, days 3, 6 and 10 post-infection. **(E)** Bacterial cell density of *E. coli*. **(F)** IP3 concentration in media from *E. coli^WT^* and *E. coli^Δphy^* cultured with 1mM phytate, per 10^8^ CFU of bacteria. **(G)** Experimental approach. **(H)** CFUs of *E. coli* in stool of monoassociated mice, normalized to sample weight, day 7 post-inoculation. **(I)** Bacterial cell density of *C. rodentium*. **(J)** CFUs of *C. rodentium* in stool of infected mice monoassociated with *E. coli^WT^* or *E. coli^Δphy^*, normalized to sample weight, days 3 and 6 post-infection. Data are representative of 2-3 independent experiments. n = 3-4 per group. Results are ± SEM. ^*^ *p* < 0.05.

Monogastric mammals such as humans and mice do not produce the phytase enzyme that breaks down phytate in the intestinal lumen (45, 46). Instead, phytate digestion is dependent on phytase that is produced by bacteria residing in the intestine. Germ free (GF) animals exhibit lower concentrations of phytate metabolites in the gut lumen as microbial phytase catalyzes the removal of phosphorus from phytate to produce phosphorous and lower forms of inositol phosphates (47–49) (**Figure 3A**). Phytate supplementation to microbiota-replete, conventionally-raised (CNV) mice increased inositol trisphosphate (IP3) concentrations in intestinal contents (**Figure 3B**), confirming that commensal microbes in the mouse intestine break down phytate. To test whether microbiota are required for the phytate-mediated defense against *C. rodentium* infection, GF mice were treated with phytate prior to infection. (**Figure 3C**). Unlike CNV mice, GF mice exhibited comparable *C. rodentium* infection between vehicle and phytate-treated groups (**Figure 3D**). Given that phytate-induced protection was lost when mice lacked commensal microbes, the microbiota are required for regulation of host defense by phytate.

These findings provoked the hypothesis that microbial-produced phytase is required for phytate-induced protection. To test this, we employed a commensal strain of *E. coli* that either expresses the wildtype phytase *AppA* gene (*E. coli^WT^*) or lacks the *AppA* gene (*E. coli^Δphy^*). To confirm phytase activity levels in these strains, *E. coli^WT^* and *E. coli^Δphy^* were cultured overnight in phytate-supplemented media, and the concentrations of IP3 in the supernatant were compared. *E. coli^WT^* and *E. coli^Δphy^* growth was similar (**Figure 3E**). However, significantly higher levels of IP3 were present in *E. coli^WT^* cultures compared to *E. coli^Δphy^* cultures, confirming impaired phytase activity in *E. coli^Δphy^* bacteria (**Figure 3F**). To next compare the role of bacterial phytase expression *in vivo*, GF mice were monoassociated with either *E. coli^WT^* or *E. coli^Δphy^* and fed phytate-containing chow (**Figure 3G**). Colonization was similar for both strains (**Figure 3H**), and phytate or phytase did not directly alter *C. rodentium* growth (**Figure 3I**). Remarkably, though, mice monoassociated with *E. coli^Δphy^* exhibited increased susceptibility to *C. rodentium* infection, relative to mice monoassociated with *E. coli^WT^* (**Figure 3J**), Therefore, phytase expressed by commensal bacteria enables dietary phytate to promote innate host defense against intestinal infection.

### Phytate consumption primes elevated epithelial antimicrobial defense

**Figure 4:**
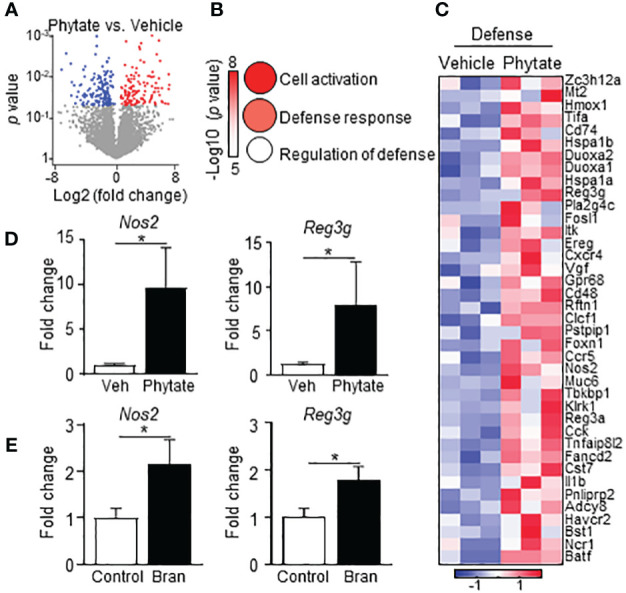
Phytate consumption primes elevated epithelial antimicrobial defense. **(A)** Volcano plot showing differentially expressed LI IEC genes between Vehicle- and 2% phytate-treated naïve mice identified by RNA-seq. Red: upregulated, blue: downregulated with phytate, *p* < 0.05. **(B)** Ontology of the genes upregulated with phytate in **(A)**. Circle sizes correspond to gene numbers in pathways. **(C)** Heatmap of relative mRNA expression of IEC defense response genes upregulated in phytate. **(D)** mRNA expression levels in LI IEC, normalized to vehicle. **(E)** mRNA expression in large intestinal IECs, normalized to control. Data are representative of 2-3 independent experiments. n = 3-4 per group. Results are mean ± SEM. ^*^ *p* < 0.05.

IECs provide initial defense against pathogenic infection in the intestine, in part through basal expression of antimicrobial molecules (50). Bran- and phytate-induced protection against *C. rodentium* occurred as early as day 3 post-infection, suggesting that phytate may regulate basal epithelial defense mechanisms. To test this hypothesis, global transcriptional profiles were compared in colonic IECs harvested from CNV mice treated with vehicle or phytate. These analyses identified numerous genes that were significantly upregulated or downregulated in IECs following phytate ingestion (**Figure 4A**). Interestingly, phytate significantly upregulated gene expression in host defense pathways. (**Figures 4B, C**). Phytate-induced defense genes included the antimicrobial peptide *Reg3g* and the bactericidal nitric oxide producer *Nos2* (**Figures 4C, D** **),** both known mediators of early defense against *C. rodentium* (51, 52). Furthermore, IEC upregulation of *Reg3g* and *Nos2* occurred in rice bran diet-fed mice compared to control mice (**Figure 4E**). Therefore, consuming rice bran or phytate induces basal epithelial antimicrobial defense mechanisms that protect against pathogenic bacterial infection in the intestine.

### Epithelial STAT3 activation in the intestine is induced by phytate

**Figure 5:**
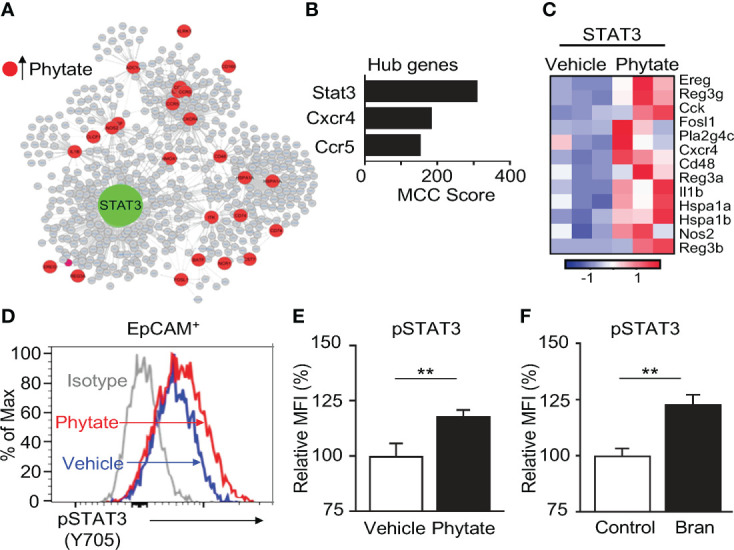
Epithelial STAT3 activation in the intestine is induced by phytate. **(A)** Interaction network of phytate-induced IEC defense genes. **(B)** Top hub genes in **(A)**. **(C)** Heatmap of relative mRNA expression of IEC STAT3-target genes upregulated in phytate-treated. **(D)** Representative flow cytometry plots from IECs of vehicle- or 2% phytate-treated mice, gated on live EpCAM^+^ cells. **(E, F)** Mean Fluorescence Intensity (MFI) of pSTAT3 (Y705), normalized to vehicle **(E)** or control **(F)**. Data are representative of 2-3 independent experiments. n=4 per group. Results are mean ± SEM. ^**^ *p* < 0.01.

To further dissect the mechanism of phytate-induced epithelial regulation, network analyses were conducted on the enriched phytate-induced defense genes. Interestingly, these analyses identified the transcription factor Signal Transducer And Activator Of Transcription 3 (STAT3) as the most central and essential factor for regulating this network (**Figures 5A, B**). Consistently, multiple known STAT3-controlled downstream genes were upregulated in IECs of phytate-fed mice (**Figure 5C**). This finding led to the hypothesis that phytate activates IEC-intrinsic STAT3. STAT3 activation is characterized by phosphorylation of its tyrosine 705 residue. Therefore, to test whether phytate alters STAT3 activation, pSTAT3 (Y705) in the large intestinal epithelium of control mice and mice receiving 2% phytate were compared. Interestingly, phytate feeding significantly increased phosphorylation of STAT3 in IECs relative to control mice (**Figures 5D, E**). Further, IECs harvested from rice bran-fed naïve mice also exhibited enhanced STAT3 activation (**Figure 5F**). Collectively, these data reveal that consuming phytate-enriched diets can increase epithelial STAT3 activation and therefore prime expression of antimicrobial targets that are regulated by STAT3.

### Phytate-mediated epithelial STAT3 activation requires HDAC3

**Figure 6:**
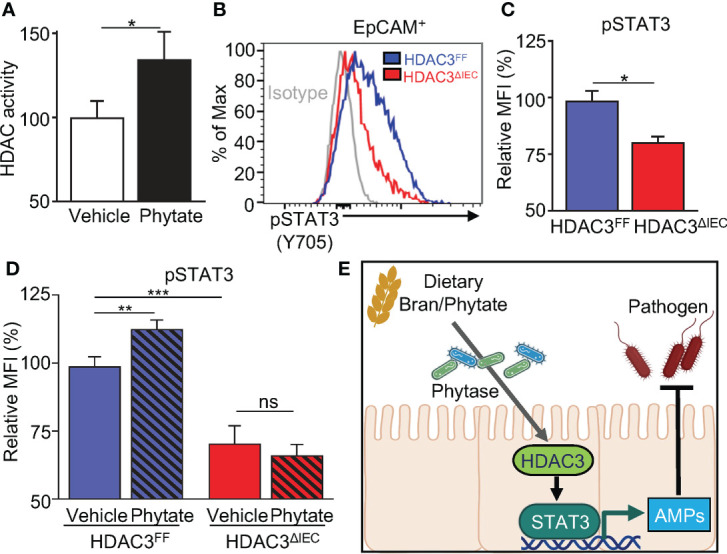
Phytate-mediated epithelial STAT3 activation requires HDAC3. **(A)** Intestinal epithelial HDAC activity of vehicle- or phytate-treated mice. **(B)** Representative flow cytometry plots from IECs of vehicle- or 2% phytate-treated mice, gated on live EpCAM^+^ cells. **(C)** Mean Fluorescence Intensity (MFI) of pSTAT3 (Y705), normalized to FF. **(D)** MFI of IEC pSTAT3 of vehicle- or 2% phytate-treated HDAC3^FF^ or HDAC3^ΔIEC^ mice, normalized to FF-vehicle. **(E)** Through phytate metabolism by microbial phytases, rice bran and its component phytate activate IEC STAT3 and downstream defense mechanisms against enteric infection. Data are representative of 2-3 independent experiments. n=4 per group. Results are mean ± SEM. ^*^ *p* < 0.05, ^**^ *p* < 0.01, ^***^ *p* < 0.001.

Deacetylation of STAT3 by the histone deacetylase HDAC3 promotes STAT3 activation in hepatocytes and lymphoma cells (53, 54). HDAC3 is an enzyme sensitive to environmental signals, and IEC expression of HDAC3 is critical in host defense against C. *rodentium* (55, 56). The phytate-derived metabolite inositol trisphosphate activates HDAC3 (47), and consistent with this, phytate-fed mice displayed increased mucosal HDAC activity relative to vehicle-fed mice (**Figure 6A**). Therefore, we first hypothesized that HDAC3 regulates STAT3 activation in IECs. To test this hypothesis, we utilized an IEC-specific HDAC3 knockout mouse model (57). Interestingly, IECs isolated from mice lacking IEC-intrinsic HDAC3 (HDAC3^ΔIEC^) displayed displayed significantly lower pSTAT3 levels relative to floxed littermate mice (HDAC3^FF^), indicating that STAT3 activation in IECs requires HDAC3 (**Figures 6B, C**). We next hypothesized that phytate-induced STAT3 activation was HDAC3-dependent. To test this, HDAC3^ΔIEC^ mice and littermate HDAC3^FF^ mice were treated with 2% phytate, and the levels of IEC pSTAT3 (Y705) were compared. The results exhibited that phytate ingestion increased STAT3 activation in IECs of HDAC3^FF^ mice, but not IECs of HDAC3^ΔIEC^ mice (**Figure 6D**). Taken together, these data demonstrate that dietary phytate mechanistically induces activation of epithelial STAT3 through regulation of HDAC3.

## Discussion

In this study, we identified that consumption of rice bran or phytate induces innate intestinal epithelial pathways that decrease susceptibility to bacterial infection (**Figure 6E**). Furthermore, this diet-induced protection is mediated by the commensal bacterial metabolism of phytate and subsequent activation of the HDAC3-STAT3 axis in epithelial cells (**Figure 6E**). Phytate-induced activation of IEC-intrinsic STAT3 required expression of HDAC3, as phytate failed to induce STAT3 activation in the absence of HDAC3 expression. Direct deacetylation of the lysine 685 residue of STAT3 by HDAC3 has been described to precede phosphorylation and nuclear translocation of STAT3 in hepatocytes (53), however, indirect mechanisms may also contribute. Similarly, inhibition of HDAC3 in lymphoma cells resulted in nuclear export of STAT3 to the cytoplasm, thus hindering STAT3 function as a transcriptional factor (54). Similar to these cells, our analyses demonstrate that loss of HDAC3 impairs STAT3 activation in IECs.

Although phytate induces HDAC3-mediated activation of STAT3 in IECs, involvement of other STAT3 activators, such as IL-22, IL-18, and IL-1β (58–60) cannot be excluded. IL-22, a well-characterized STAT3 activator, is produced by several different immune cells in the intestine in response to cytokines IL-6 and IL-23, and aryl hydrocarbon receptor (AhR) ligands (51, 61, 62). Further, vitamin A, tryptophan metabolites, and short-chain fatty acids have been shown to induce IL-22 production from intestinal immune cells (63–65). In addition, IECs can propagate the immune cell IL-22 response through secretion of chemoattractant resistin-like molecule-beta (RELM-β) that recruits IL-22 producing CD4^+^ T cells to the sites of infection (66). Thus, beyond epithelial-intrinsic regulation, future studies will be necessary to determine the contribution of phytate to local and systemic immune cell responses that promote host defense. This will include extending the analyses of phytate effects on immune follicles and mucin-producing goblet cells (66), as well as additional sites such as the small intestine and extra-intestinal tissue.

Constituents of the microbiota can alter susceptibility to pathogenic *C. rodentium* infection through direct inhibition *via* nutritional competition or production of bacteriostatic molecules such as butyrate and sulfide (13, 67, 68). Phytate supplementation did not result in a significant deviation of microbiota composition or luminal SCFAs concentrations compared to vehicle-supplemented mice in dextran sulfate sodium (DSS)-treated mice (47). On the other hand, increased *Lactobacillus* spp. has been reported with phytate supplementation in high sucrose-fed rats (69). Increased levels of *Lactobacillus* spp. have also been reported with 4-week consumption of 10% bran diet in rodents (70). Microbial phytases encompass multiple classes of enzymes and are expressed by a variety of commensal bacterial and fungal species in the intestinal lumen (71). Our gnotobiotic model employing a phytase-deficient commensal strain demonstrated that microbial phytase was essential in protection against infection, verifying the importance of phytate and its metabolites in the regulation of host epithelial defense. Phytase deficiency in commensal *E. coli* did not result in growth disadvantage in the monocolonization model in our study. However, it is possible that commensal bacterial phytase-derived products also directly alter *C. rodentium* virulence and therefore not only promote protection *via* regulation of host immunity. Elucidating the association between phytase abundance and individuals’ responsiveness to dietary phytate may be essential for guiding the use of phytate for nutritional intervention strategies.

The data presented in this manuscript reveal a new mechanism of dietary regulation of mucosal immunity in which metabolism of phytate-rich foods by commensal bacteria primes epithelial defense against infection *via* an HDAC3-STAT3 pathway. Evidence also suggests that rice bran decreases susceptibility to other enteric pathogens, such as *Salmonell*a and rotavirus (40, 72). Therefore, consuming bran and other phytate-enriched foods may represent an effective dietary strategy for broadly boosting innate mucosal defense against multiple intestinal pathogens. Discovery of this diet-microbiota-host regulatory mechanism provides fundamental insights that can guide practical and personalized nutritional interventions designed to enhance mucosal immunity.

## Methods

### Mice

All murine experiments were performed according to guidelines of the Institutional Animal Care and Use Committee. Animals were housed up to 4 per cage in a ventilated cage with 12 h light/dark cycle and free access to chow and water. Animals were provided with appropriate care by a licensed veterinarian. Floxed Hdac3 mice were bred to C57BL/6 mice expressing Cre-recombinase under the control of villin promoter to generate HDAC3^ΔIEC^ mice (57). Gnotobiotic mice were maintained in sterile isolators (Class Biologically Clean) in the CCHMC Gnotobiotic Mouse Facility, fed autoclaved food and water, and routinely monitored to ensure the absence of microbial contamination. To establish *E.coli* and phytase-KO *E. coli* monoassociated mice, GF mice received 1 x10^9^ CFU bacteria in PBS *via* oral gavage. Monoassociated mice were housed on a sealed positive pressure IVC rack (Allentown). Phytate (phytic acid sodium salt hydrate, Sigma) was dissolved in water to make 2% phytate and filtered (0.22μm) prior to providing to the mice as drinking water. Control diet (TD.160791) or 20% rice bran (NOW stabilized rice bran) diet (TD.210517) were custom made by Envigo with matched macronutrients, calories, minerals, vitamins, and fiber. Mice were infected with 1x10^9^ CFU GFP-*C. rodentium* (DBS100) *via* oral gavage. Stool and colon tissues were homogenized in sterile PBS using a Tissue Lyser II, serially diluted and plated on MacConkey agar. CFUs were counted and normalized to stool weight after 16 hr. For histologic analyses, sections of colon were fixed in 10% neutralized formalin, paraffin embedded, sectioned, and stained with hematoxylin and eosin.

### IEC isolation and RNA analyses

IECs were isolated from the large intestine by shaking tissue in 1mM EDTA/1mM DTT 5% FBS PBS at 37°C for 10 min as described previously (57). RNA was extracted from cells using the RNeasy Kit (Qiagen) according to manufacturer’s instructions. For RT-qPCR, RNA was reverse-transcribed with Verso reverse transcriptase (Invitrogen) and expression was compared using SYBR (Applied Biosystems) and analyzed in the linear range of amplification. Target gene expression was normalized to an unaffected control gene. For global expression analyses, 3 biological replicates of IECs from vehicle- and phytate-treated mice were compared. Reads were bar codes trimmed and mapped to mouse genome (GRCm38) using Bowtie2. The reads aligning to known transcripts were quantified using Seqmonk (V1.47.1) and visualized using Genepattern Multiplot Studio. Differential expression analysis was performed using EdgeR within Seqmonk (p<0.05, fold change >1.5). For pathway and ontological analyses, gene lists were submitted to the Toppgene database (toppgene.cchmc.org), which amasses ontological data from over 30 individual repositories. Network construction and identification of hub genes using Cytohubba were done on Cytoscape v3.9.1.

### Flow cytometry

IECs were isolated as described above. Cells were stained using the following fluorescence-conjugated monoclonal antibodies diluted in FACS buffer (2% FBS, 0.01% sodium azide, PBS): Brilliant Violent 711 anti-CD326 (EpCAM) (Clone: G8.8, BD Biosciences), PE anti-pSTAT3 (Tyr 705) (Clone: LUVNKLA, Invitrogen), PE anti-Mouse IgG2bκ isotype (Clone: eBMG2b, Invitrogen). Dead cells were excluded with the Violet dead Cell Stain Kit (Invitrogen). For pSTAT3 staining, the cells were fixed in 4% PFA following dead cell staining, then permeabilized with methanol prior to surface and pSTAT3 or isotype staining. Samples were acquired on the Canto III and analyzed with FlowJo™ v10.8 Software (BD Life Sciences).

### IP3 assay

Fecal pellets were homogenized in cold PBS and extract was collected after centrifugation at 4°C. IP3 ELISA was performed on fecal extract or bacterial culture supernatant according to manufacturer instructions (MyBiosource). Briefly, samples were incubated with 50 µl of biotinylated detection antibody for 45 minutes at 37°C, washed 3 times, and incubated with HRP conjugate at 37°C for 30 minutes. The plate was rinsed with wash buffer followed by substrate incubation for 15 minutes at 37°C. The reaction was stopped, and the optical density of each well was measured using a micro-plate reader (Biotek Synergy 2) set to 450 nm.

### Bacterial culture and quantification

Wild type commensal *E. coli* K-12 (ATCC 700926) and phytase-KO *E. coli* CU-1867 (ATCC 47092) colonies were grown on LB agar plate and inoculated in 10 ml LB broth for overnight culture prior to administration to GF mice in mono-association studies. For culture media IP3 quantification study, *E. coli* were cultured with 1 mM phytate. Mono-associated mice were monitored for contamination by quantitative PCR. Fecal samples were collected in 2ml pre-weighed sterile microcentrifuge tubes. Fecal bacterial DNA was isolated using QIAamp^®^ Fast DNA Stool Mini Kit (Qiagen) following the kit protocol. Bacterial DNA was assessed by quantitative PCR (QuantStudio3; Applied Biosystems) using 16S-rRNA and bacterial-specific primer pairs (Invitrogen, MilliporeSigma). PCR of *E. coli* phytase gene (*AppA*) was performed to confirm lack of *AppA* in phytase-KO *E. coli* (47). To determine CFUs in mono-associated mice, stool was homogenized, serially diluted, and grown on Lennox LB plates in aerobic condition overnight. CFUs were normalized to stool weights.

### HDAC activity

LI mucosa was lysed in RIPA buffer and HDAC activity was assayed using a fluorometric assay (Active Motif). Briefly, 10 μg of cell lysate was incubated with 100 μM of HDAC substrate at 37°C for 1 hour. 50 μl of developer solution containing 2 μM of trichostatin A (TSA) was added at room temperature to stop the reaction. Fluorescence was measured using a fluorescent plate reader (Biotek Synergy 2) with an excitation wavelength of 340 nm and an emission wavelength of 460 nm.

### Statistics

All statistical analyses were performed using GraphPad Prism 8.0. Statistical significance was determined by students t-test or ANOVA. All data meet the assumptions of the statistical tests used. Results are shown as mean ± SEM and considered significant at *p*<0.05 (*); *p*<0.01 (**); *p*<0.001 (***).

## Data availability statement

The original contributions presented in the study are included in the article/supplementary materials. Further inquiries can be directed to the corresponding author.

## Ethics statement

The animal study was reviewed and approved by Cincinnati Children’s Institutional Animal Care and Use Committee.

## Author contributions

TA and SH-H designed the studies and analyzed the data. SH-H, LC, VW, LE, SA, and JW carried out experiments. TA and SH-H wrote the manuscript. All authors contributed to the article and approved the submitted version.

## Funding

This research is supported by the National Institutes of Health (DK114123, DK116868 to TA). TA holds a Kenneth Rainin Foundation Award and Pathogenesis of Infectious Disease Award from the Burroughs Wellcome Fund. This project is supported in part by PHS grant P30 DK078392.

## Acknowledgments

We thank the Way, Qualls, and Deshmukh labs for useful discussions and members of the Alenghat lab for critical reading of the manuscript. We thank CCHMC Veterinary Services, Research Flow Cytometry, and Pathology Research Core for services and technical assistance.

## Conflict of interest

The authors declare that the research was conducted in the absence of any commercial or financial relationships that could be construed as a potential conflict of interest.

## Publisher’s note

All claims expressed in this article are solely those of the authors and do not necessarily represent those of their affiliated organizations, or those of the publisher, the editors and the reviewers. Any product that may be evaluated in this article, or claim that may be made by its manufacturer, is not guaranteed or endorsed by the publisher.
